# Supervised internship in undergraduate nursing courses in the State of São Paulo, Brazil*

**DOI:** 10.1590/1518-8345.3540.3288

**Published:** 2020-06-08

**Authors:** Larissa Sapucaia Ferreira Esteves, Isabel Cristina Kowal Olm Cunha, Elena Bohomol

**Affiliations:** 1 Universidade Federal de São Paulo, Escola Paulista de Enfermagem, São Paulo, SP, Brazil.; 2 Universidade do Oeste Paulista, Faculdade de Enfermagem, Presidente Prudente, SP, Brazil.; 3 Scholarship holder at the Coordenação de Aperfeiçoamento de Pessoal de Nível Superior (CAPES), Brazil.

**Keywords:** Teaching, Learning, Training Support, Education, Nursing, Education, Higher, Schools, Nursing, Ensino, Aprendizagem, Apoio ao Desenvolvimento de Recursos Humanos, Educação em Enfermagem, Educação Superior, Escolas de Enfermagem, Enseñanza, Aprendizaje, Apoyo a la Formación Profesional, Educación en Enfermería, Educación Superior, Facultades de Enfermería

## Abstract

**Objective:**

to analyze how nursing courses in the State of São Paulo, Brazil have operationalized the supervised curricular internship and to identify those that approach the recommendations proposed by the National Curriculum Guidelines.

**Method:**

a quantitative, descriptive-exploratory study. The sample consisted of 38 course coordinators. The data collection instrument was developed based on the Curricular Guidelines. Data collection took place electronically and, for data analysis, descriptive and inferential statistics were used.

**Results:**

the undergraduate schools have developed internships for a mean of 860.4 hours in primary and tertiary care settings, with learning based on professional practice being the main teaching method. Formative assessment is the predominant mode of assessment, and nurses from health institutions participate in 44.7% of courses. The mean score obtained was 3.1 points (scale from 1 to 5), with the evaluation processes used being the most influential factor (p<0.001).

**Conclusion:**

the courses have partially met the educational legislation regarding the hours and participation of professionals from health institutions granting internship field, which can compromise the quality of training and the safety of care.

## Abstract (pt)

**Objetivo:**

analisar como os cursos de enfermagem no Estado de São Paulo, Brasil têm operacionalizado o estágio curricular supervisionado e identificar os que se aproximam das recomendações propostas pelas Diretrizes Curriculares Nacionais.

**Método:**

estudo quantitativo, descritivo-exploratório. A amostra foi composta por 38 coordenadores de curso. O instrumento de coleta de dados foi elaborado a partir das Diretrizes Curriculares. A coleta de dados ocorreu por meio eletrônico, e para a análise de dados utilizou-se a estatística descritiva e inferencial.

**Resultados:**

as escolas de graduação têm desenvolvido o estágio por meio de 860,4 horas, em média, em cenários de atenção básica e terciária, sendo a aprendizagem baseada na prática profissional o principal meio de ensino. A avaliação formativa é o modo predominante de avaliação, e há a participação dos enfermeiros das instituições de saúde em 44,7% dos cursos. A pontuação média obtida foi de 3,1 pontos (escala de 1 a 5), sendo os processos de avaliação utilizados o fator de maior influência (p<0,001).

**Conclusão:**

os cursos têm atendido parcialmente a legislação educacional quanto à carga horária e participação dos profissionais das instituições de saúde concedentes de campo de estágio, o que pode comprometer a qualidade na formação e a segurança dos cuidados.

## Abstract (es)

**Objetivo:**

analizar de qué manera los cursos de enfermería en el Estado de San Pablo, Brasil, han puesto en práctica la pasantía curricular supervisada e identificar aquellos que se acercan a las recomendaciones propuestas por las Directrices Curriculares Nacionales.

**Método:**

estudio cuantitativo, descriptivo-exploratorio. La muestra consistió en 38 coordinadores de cursos. El instrumento de recolección de datos se desarrolló en base a las Directrices Curriculares. La recolección de datos se realizó electrónicamente y para el análisis de datos se utilizaron estadísticas descriptivas e inferenciales.

**Resultados:**

las instituciones de títulos de grado han desarrollado pasantías por una media de 860,4 horas en entornos de atención primaria y terciaria, siendo el aprendizaje basado en la práctica profesional el método principal de enseñanza. La evaluación formativa es el modo predominante de evaluación, y los enfermeros de las instituciones de salud participan en el 44,7% de los cursos. El puntaje medio obtenido fue de 3,1 puntos (escala de 1 a 5), siendo los procesos de evaluación utilizados el factor más influyente (p<0,001).

**Conclusión:**

los cursos han cumplido parcialmente la legislación educativa sobre la carga horaria y la participación de profesionales de instituciones de salud que otorgan pasantías, lo que puede comprometer la calidad de la capacitación y la seguridad de la atención.

## Introduction

Discussions and reflections about the direction of Nursing Education have intensified in the last two decades, driven mainly by economic and social changes worldwide^(^ 1 ^)^. The effectiveness of clinical teaching for the training of nurses, their individualization, pedagogical innovations, the participation of professionals from health institutions that receive students in internship situations and the satisfaction of students with their learning have been questioned by researchers^(^ 2 ^-^ 3 ^)^.

In Brazil, the training of nurses has been widely debated by educational institutions, bodies representing the class and the government, which has generated numerous opinions and resolutions. Several points of disagreement have been elucidated over the years, however, other problems seem to generate greater discomfort, such as the development of Supervised Curricular Internship (*Estágio Curricular Supervisionado*, ECS) in health services^(^ 4 ^)^.

Higher Education Institutions (HEIs) organize their training processes based on the National Curriculum Guidelines (*Diretrizes Curriculares Nacionais*, DCNs) for undergraduate nursing education. This establishes the desired professional profile, its essential competences and determines the minimum curricular structure, in which the student must take theoretical and practical disciplines throughout the training and, at the end, 20% of the total hours of the undergraduate course must be directed to the development of the ECS^(^ 5 ^)^.

The ECS has a central role in the training of nurses, because it aims to give the future professional greater mastery of the practice, articulation of knowledge and fundamental actions to instrumentalize the working process of the profession^(^ 6 ^)^. In addition to presenting itself as an integrating instrument between the HEIs and the Health Institutions, it is a curricular component that allows for the insertion of the student in the real world of work, mediated by the figure of the teacher and tutored by the professional nurse who works in the scenario where the teaching practices^(^ 4 ^-^ 6 ^)^.

It is possible to state that the nurse is the professional who can guarantee the sustainability of universal health systems, given the expertise in care and the ease in inter-professional relationships. Training processes that guarantee theoretical knowledge and, especially, mobilization of cognitive skills in professional practice can guarantee an individual-centered approach, leadership, technological skills, effective communication with social participation, practice improvement processes, safety, teamwork and cooperation, care coordination and quality improvement^(^ 7 ^)^.

The ECS is configured as a strategy that aims to insert the professional future in the contexts where the profession is developed, without filters or controls, as it is presented, guaranteeing training for the mobilization of cognitive, psychomotor and affective skills necessary to work in complex health systems^(^ 3 ^,^ 8 ^-^ 9 ^)^. Nursing students from member countries of the European Union spent about half of their time in clinical practice, which is equivalent to about 2,460 hours, as it is considered that training for professional training requires direct contact with the patient in clinical practice. To guarantee basic knowledge and skills to adhere to clinical practice scenarios in health services, students go through theoretical activities and significant hours of simulated training^(^ 10 ^)^.

Thus, it can generate an important contribution, as it is configured as a strategy with a real impact on the transformation of professional training when inserted in an expanded curricular structure, which guarantees both clinical practices of specific disciplines and moments aimed at the integration of teaching-service-community in diversified scenarios^(^ 9 ^)^.

However, a study shows that there are few national publications that portray the way in which Brazilian nursing schools operate their ECS, what have been the hours devoted to this moment of training, if there is active participation of nurses who receive students in the internship fields and also if nursing schools have performed ECS or just clinical practices at the end of graduation^(^ 6 ^)^.

Thus, this study aims to analyze how nursing courses have operationalized ECS in the state of São Paulo, Brazil, and to identify those that approach the recommendations proposed by the DCNs to undergraduate courses.

## Method

A study with a quantitative and descriptive-exploratory approach, carried out from July 2016 to July 2017. The research universe consisted of undergraduate courses, registered with the Ministry of Education (*Ministério da Educação*, MEC) in the presential modality, allocated in the state of São Paulo, Brazil. The sampling method was non-probabilistic for convenience since, in 2016, there were 190 nursing course registrations in the e-MEC System^(^ 11 ^)^ and, of these, 27 were excluded because they had double register. The population consisted of the coordinators of 163 nursing bachelor’s courses, and 38 (23.3%) courses comprised the research sample.

The data collection instrument was developed by the researchers, using the DCNs for undergraduate nursing courses^(^ 5 ^)^ as a theoretical framework. It is self-applied, with 13 items distributed in two parts. The first includes three variables used to obtain information about the HEI: administrative dependency (public or private), characteristics of the course in terms of duration in years and the total hours. The second part was composed of 10 variables containing multiple choice and open questions, identified by the letter P (question, “pergunta” in Portuguese): P1: specific hours for the ECS, P2: moment of the course when the ECS starts, P3: existence of simulated practice in the laboratory before starting the ECS and total hours, P4: existence of supervised clinical practice in health services before starting the ECS and total hours, P5: practice scenarios used for the development of the ECS, P6: forms of teacher supervision, P7: teaching-learning means and methods employed, P8: learning assessment processes, P9: existence of the participation of nurses working in the granting units in the planning of the ECS and the teaching-learning process and P10: strategies for teaching-service integration.

Before starting data collection, the instrument underwent an evaluation process with eight experts in order to verify the clarity and relevance of the questions. The body of evaluators was made up of professors and course coordinators from public and private universities, professionals involved with class representation bodies and nurses who received internship students. The selection was intentionally carried out by the research team, after analyzing the Lattes Curriculum that provided the recognition of interfaces between the evaluator and the researched theme. The invitation to participate in the research was made via telephone contact and after acceptance, the instrument was sent via e-mail.

The data collection procedure took place online, between August 2016 and November 2017. Based on the electronic system of the Ministry of Education^(^ 11 ^)^, a spreadsheet was prepared using the Microsoft Excel Program^®^, containing the name of the HEI of each registered undergraduate course and the electronic addresses that gave access to the official website of the institution, which were visited one by one to obtain the full name of the coordinator, his electronic address and institutional telephone number. Each coordinator received, via e-mail, the letter of introduction, which contained the invitation to participate in the study, a brief explanation of the research and provided the electronic address to access the website of the research. On the website of the research, the participant obtained the objectives of the work, the research project in its entirety and the Free and Informed Consent Term (FICF). After signing, the respondent received, via email, a login and password, generated randomly by the system, giving access to the data collection instrument.

To identify the courses that came closest to the DCNs, regarding the development of the ECS, after obtaining the data the second part of the instrument was scored in order to obtain a ranking among the courses. One point was assigned to each question (P.1 to P.10). The questions that had only one answer (P.1, P.2, P.3, P.4, P.9) received a maximum score of one point when they stated positively (agreement with the DCNs). In the questions with the possibility of more than one answer (P.5, P.6, P.7, P.8, P.10), the score was divided by the number of alternatives that each question had, and the higher the number of resources used in ECS teaching, the higher the score. After assigning the scores, the research team requested a new assessment from the group of experts before data collection and from the statistical professional who composed the research. The maximum score awarded was 10 points, which was converted to a scale of 0 to 5, following the same pattern as for national graduation education assessments.

The results were organized, tabulated and analyzed using the *Statistical Package for the Social Sciences* (SPSS) software, version 23.0. Descriptive and inferential statistics were used to systematize and summarize the information, including frequencies, measures of central tendency and variability. Inferential analysis was performed using the *Student* t test and linear regression analysis. Both made it possible to test the significance of the regression coefficients used to identify the questions (variables) that influenced the results of this research. A model with an adjusted R square of at least 0.80 was considered as a significant model. It was considered as statistically significant when the probability was less than or equal to 5% (p<0.05). The work was approved by the Research Ethics Committee Involving Human Beings of the Federal University of São Paulo (CAAE 43875415.1.0000.5505).

## Results

The sample consisted of 38 (23.3%) undergraduate nursing courses, 32 (84.2%) of which were from the private sector. The training time for undergraduate nursing in the state of São Paulo ranged from four to six years, with 18 (47.4%) undergraduate courses developing their training processes in four years. As for the total hours of the courses, it was observed that there was a variation between 4,000 to 6,000 hours, with a mean training time of 4,304.5 hours (SD=455.4 hours). The hours devoted to the development of the ECS varied from 400 to 1,500 hours, with a mean of 860.4 hours (SD=155.9 hours). With regard to the student’s preparation time to enter the ECS, 28 (73.7) courses use a mean time of 221.9 hours (SD=205.7 hours) for simulated practices and, on average, 508.1 hours (SD=385.2 hours) for clinical practices performed in health services (Table 1).

**Table 1:** Total hours of undergraduate nursing courses in the state of São Paulo, supervised curricular internship, practical activities in simulation laboratories and practical activities in health services. São Paulo, SP, Brazil, 2017

Description	n.	Mean	Median	Standard	Minimum	Maximum
Total class hours	38	4304.5	4066.0	455.4	4000	6000
ECS* hours	36	860.4	827.0	155.9	400	1500
Class hours of the Simulated Practice	28	221.9	160.0	205.7	12	800
Class hours of the Clinical Practice	26	508.1	400.0	381.2	50	1500

* ECS = Estágio Curricular Supervisionado (Supervised Curricular Internship)

Table 2 presents the data referring to the scenarios used for ECS, forms of teacher supervision, means and teaching-learning methods developed, and types of assessment carried out during the ECS. It is observed that the main scenarios were the following: General hospitals, cited 35 times (92.1%), and Family Health Units, cited 34 times (89.5%).

**Table 2:** Description of the scenarios, forms of teacher supervision, main means and methods of teaching-learning and form of evaluation used by undergraduate nursing courses in the state of São Paulo in the development of the Supervised Curricular Internship. São Paulo-SP, Brazil, 2017*

Scenarios used as a field	n	%
General Hospital	35	92.1
Family Health Strategy	34	89.5
Basic Health Unit	33	86.8
Specialties Ambulatory	25	65.8
Elementary and/or High School	15	39.5
Long-term care facility for the elderly	15	39.5
Rehab	9	23.7
Laboratories	5	13.2
Technical schools	1	2.6
**Means and methods of teaching and learning used**	**n**	**%**
Learning based on the professional practice	33	86.8
Clinical case studies	32	84.2
Problem-based learning	20	52.6
Seminars	18	47.4
Problematization	16	42.1
Portfolios	15	39.5
Simulated practice laboratories	13	34.2
Conceptual maps	10	26.3
Learning cycle development	7	18.4
Workshops among students, teachers and field professionals	5	13.2
Dramatization	5	13.2
Presential or virtual forums	4	10.5
**Learning assessment processes**	**n**	**%**
Formative evaluation	28	73.7
Subjective evaluation performed by the teacher	27	71.1
Subjective self-evaluation	26	68.4
Subjective evaluation performed by the field nurse	22	57.9
Summative assessment (tests or formal evidence)	18	47.4
Evaluation in simulated practice scenarios	9	23.7
Valuation through portfolio	1	2.6
**Forms of supervision from the teacher accompanying the student**	**n**	**%**
Direct supervision	27	71.1
Indirect supervision	23	60.6
**Nurses’ participation in the elaboration and development of the internship plan**	**n**	**%**
Yes	17	44.7
Partially	16	42.1
No	3	7.9
**The course develops strategies to integrate teaching and health service**	**n**	**%**
Yes, with all internship fields.	18	47.4
Yes, with some internship fields.	13	34.2
No	5	13.2

* The questions allowed for more than one answer

As for the teaching-learning means and methods used in the development of the ECS, for 33 (86.8%) schools the process occurs through learning based on professional practice, inserting the student in the health establishment autonomously, mediated by the nurse and teacher. Regarding the form of student assessment, the courses use more than one strategy, with formative assessment being the most cited (73.7%), and, subsequently, the subjective assessment carried out by the teacher in 27 (71.1%) courses.

Regarding the forms of supervision, 27 (71.1%) courses claim to perform direct supervision, in which the teacher remains with the student in the internship field throughout the period; 23 (60.6%) perform indirect supervision, in which the student remains in the health institution following the activities developed by the service nurse and the teacher is present at pre-established moments inside or outside the internship unit. As for the nurse’s participation in the development of the ECS and the teaching-service integration, 17 (44.7%) schools affirm that there is the participation of the professional in the planning of the ECS and during the teaching-learning process, and 18 (47.4 %) develop strategies that aim to integrate teaching-service in all fields where ECS is held. Among the strategies used to bring the educational and health institution closer together, the participation of nurses in the students’ evaluation processes stands out, strategies that make nurses propose changes in the ECS plan and participate in the planning of internship actions.

**Figure 1:**
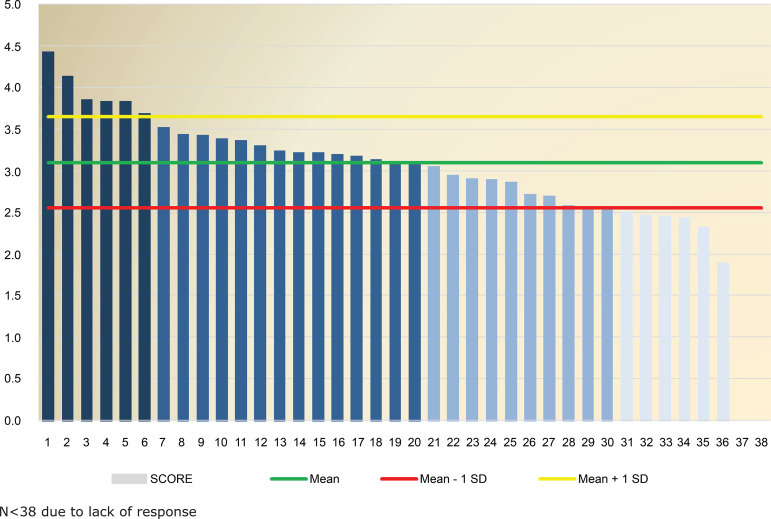
Ranking of the score obtained by undergraduate courses in the state of São Paulo participating in the research. São Paulo-SP, Brazil, 2017 N<38 due to lack of response

Figure 1 shows the ranking of the score of nursing courses that participated in the research. It is noticed that the maximum score obtained was 4.43 points, with a mean of 3.10 points (SD=0.55), upper limit (mean + standard deviation upwards) 3.65 and the lower limit (mean + standard deviation downwards) 2.55. It was found that six (15.78%) courses are above the upper mean, and six (15.78%) scored below the lower limit.

The linear regression analysis identified the questions that influenced the score obtained by the undergraduate courses. Those that obtained the highest coefficient were the following: P8, which identified the learning assessment processes used during the ECS with a regression coefficient of 0.93 and p<0.001; P7 was about the teaching-learning methods and methods used in the course with a regression coefficient of 0.77 and p=0.007; P2 that identified the moment of the course when students enter the ECS with a regression coefficient of 0.66 and p=0.02; P10 identified whether the course promoted teaching-service integration strategies; P3 who verified that the courses held simulated practical classes in a skills laboratory before the start of the ECS with a regression coefficient of 0.47 and p<0.001, and, finally, P4 raised data about the existence of supervised clinical practice in health services before starting the ECS with a regression coefficient of 0.39 and p=0.001. Table 3 shows the linear regression, t test and identification of the level of significance used to analyze the influence of the variable in the results obtained.

**Table 3:** Linear regression analysis, application of the t test and identification of the level of significance of the variables studied. São Paulo, SP, Brazil, 2017

Variables	Coefficients	T statistics	Significance	95.0% Confidence Interval for the Coefficient	
				Lower limit	Upper limit
P8	0.93	4.30	<0.001	0.49	1.38
P7	0.77	2.93	0.007	0.23	1.31
P2	0.66	3.46	0.002	0.27	1.05
P10	0.57	4.44	<0.001	0.31	0.83
P3	0.47	4.67	<0.001	0.27	0.68
P4	0.39	3.84	0.001	0.18	0.60

## Discussion

In its majority, the sample of the present study was composed by private schools. The 2016 Census of Brazilian Higher Education^(^ 12 ^)^ shows that 87.7% of the higher education institutions are private and together account for 75.3% of all the enrollments in graduation courses. In the state of São Paulo, universe of this study, the proportion of students in on-site undergraduate courses in the private network is higher than the national mean. Such data is second only to Chile, which since the early 1980s has no longer offered public undergraduate education^(^ 13 ^)^. National studies have shown that there has been an increase in private institutions in the last ten years, with a growth of 122%^(^ 14 ^)^.

This study showed the variability of hours both for the course as a whole and for the hours devoted to the development of the ECS. For nursing education in Brazil, a minimum of 4,000 hours^(^ 5 ^)^ is determined, and this requirement is met according to the mean found.

Regarding the hours of nursing courses developed in Europe, it is observed that, although there is a diversity of curricula, in most of the higher nursing schools, the student, in order to complete the first training cycle (degree), takes three to four years of formal education, and the minimum hours must not be less than 4,600 hours. It should be noted that 50% of this hours should be devoted to clinical teaching, that is, clinical practice in health units^(^ 4 ^,^ 15 ^-^ 16 ^)^, more than double the ECS hours required in Brazil^(^ 5 ^)^. However, the offer of clinical practices is also a challenge for the European countries, because the number of hours devoted to the clinical practice has varied between 30 and 60% of the total course load, showing the non-compliance with European legislation^(^ 16 ^)^.

The minimum time for the training of nurses is established in order to guarantee capable professionals to work in the health sector in a dynamic and autonomous way, as it is an area that undergoes constant changes and advances in the area of knowledge. However, nurses working in the labor market considered that the training received does not meet the needs of their work activity, which formalizes the need to rethink the quality and hours offered by undergraduate nursing courses, today. There is a consensus that the hours for training are directly proportional to the improvement in the quality of patient care and the reduction of adverse events^(^ 17 ^-^ 18 ^)^.

The hours for the training of nurses are distributed by the DCNs in various teaching strategies. In the initial years of the course, the student is instrumentalized through theoretical classes, simulated practices and clinical practices and there is no clear definition of the hours that should be allocated to this type of education^(^ 5 ^)^. What has been observed in the state of São Paulo is the shrinking of the hours of practical classes, both simulated and clinical, since both are developed in 730 hours, on average. Thus, less than 20% of the total hours is devoted to the development of professional skills in the nursing courses surveyed, showing that students may be unprepared for the development of care skills.

Clinical nursing education aims to develop professional skills based on the theoretical knowledge acquired and the development of personal characteristics, such as the ability to reflect to act effectively with competence. Clinical learning is considered a vital part of the process, and can have a huge impact on learning experiences, in addition to influencing students’ confidence, their sense of belonging and respect for the user^(^ 4 ^,^ 16 ^)^.

Data from this study showed that, although 860.4 hours, on average, are dedicated to the development of ECS activities, there were courses that offered less than half the hours determined by the DCNs^(^ 5 ^)^. The reduction of the student’s contact time with the nurse’s work practice compromises the development of minimum professional skills for the implementation of safe and quality health practices, considering that this strategy aims to submerge the student in the real world of work, in an action-reflection-action process^(^ 6 ^)^. The ECS, developed in the last two semesters of undergraduate nursing, assumes the character of a terminal activity for the synthesis of training, validating the training process. Among the difficulties in implementing the ECS, it is observed that the integration of academy-service within and outside the walls is still incipient and that there is a strong dissociation between theory and practice^(^ 14 ^)^.

As for the places where the activities of the ECS are operationalized, it is noted that the two major scenarios of the SUS are being contemplated: primary and hospital care. In European countries, to be recognized as a field of clinical learning, health institutions must demonstrate specific criteria related to facilities, including equipment and human resources, the quality of the educational environment determined through audits and positive feedback received from independent accreditation bodies, which monitor the entire nursing education process^(^ 16 ^)^.

It was observed that, for the development of the ECS, the undergraduate courses in São Paulo have diversified their teaching-learning methods and guaranteed more flexible assessment processes with regard to the development of competences, this being the second factor with the greatest influence on the results of this research. The main changes that have occurred in the Brazilian courses aim to introduce active teaching-learning methodologies and formative assessment, making the student the center of the teaching-learning process. In line with the competences to be developed, there is a strong incentive for the construction and implementation of integrated curricula, development of actions that can improve the integration of theory and practice and also the teaching-service relationship, in addition to ensuring diversification of the learning scenarios in continuous approximation of the world of education with the world of work. However, any curricular experience must be characterized by ample exposure to excellent models of general orientation, development of skills that can guarantee the resolution of common health-disease processes^(^ 14 ^,^ 19 ^)^.

In Brazil, there are few studies describing teaching-learning methods and methods used with students in internship situations. Active methods aim to ensure meaningful learning for students, but do not direct how their supervision should occur, both by the teacher and by professionals from health institutions. The Clinical Supervision methodology, used outside Brazil, has proven to be a robust tool when the objective is to support the development of cognitive, psychomotor and affective skills. It aims to direct and organize the teacher-student-professional teaching triad, being an interesting alternative to compose the teaching methods employed^(^ 4 ^)^.

Emphasizing the potential of evaluation in the regulation of the quality of teaching and, consequently, of professional training, it was observed that in this study the question that had the greatest impact on the results obtained by the participating courses was about the processes of evaluation of learning used during the ECS. The assessment of student learning is a planned, systematized resource, which aims to obtain and analyze data on students’ knowledge and skills, which can enable the measurement of teaching processes and generate triggers for improvement actions^(^ 20 ^)^.

The teaching models that presuppose learning based on the professional routine imply the use of evaluations as a process of self-regulation of learning, carried out through the use of various instruments capable of obtaining information through non-standardized observation of the tasks and activities performed. Therefore, such processes consider the various actors who participated in professional training, including the student himself, as the center of teaching and learning activities^(^ 21 ^)^.

Less than half of the participants in this study clearly stated that there is the participation of the professional in the ECS activities, being reinforced by nursing courses when they declare that students are directly supervised by the teacher. The participation of nurses from the health institutions in training processes has been a controversial subject and, historically, difficult to be transposed^(^ 22 ^)^. The DCNs describe the need for the effective participation of professionals in the programming and in the student supervision process in the health services where the internships^(^ 5 ^)^ are developed. However, the Federal Nursing Council allows nurses to participate in planning and supervising students in the ECS activities^(^ 22 ^)^. It is considered that the nurse has a fundamental role in the insertion of the student in the internship fields, as it raises discussions about the problems experienced in professional practice, in addition to being the profession itself being carried out concretely, ensuring more significant learning^(^ 19 ^)^. In other countries, the nurse actively participates in the training processes, acting as preceptor of undergraduate students, which gives him the autonomy to plan with the teachers the activities that will be developed, supervise the development of professional skills and actively participate in the evaluation processes^(^ 22 ^)^. It is a consensus among the European nursing schools that clinical teaching is not carried out without the participation of nurses, as they encourage self-criticism, self-supervision, and reflection on work processes^(^ 4 ^)^.

The delay in Brazilian nursing education is noteworthy, which still questions the participation of the assisting nurse himself in the training process of his peers, while it should be already incorporating other professionals who make up the health team with the objective of creating teaching-learning strategies that could overcome the historical fragmentation of work and its implications for the quality of health care and patient safety. Traditional education with uni-professional practices restricts the effective attendance of complex health needs, as well as the operationalization of the principles adopted by the SUS. In this sense, the urgency lies in the need to move towards teaching processes that foster Interprofessional Education (*Educação Interprofissional*, EIP) by bringing elements capable of reversing the logic of vertical education, with a view to promoting shared learning, enabling advances in the process of the work of health teams present in the Brazilian reality^(^ 23 ^)^.

In Brazil, undergraduate nursing courses undergo cyclical assessment processes through the National Student Performance Exam (*Exame Nacional de Desempenho do Estudante*, ENADE), one of the pillars of the evaluation of the National Undergraduate Education Assessment System (*Exame Nacional de Desempenho do Estudante*, SINAES). The results of these evaluation instruments allow to know the way of functioning and the quality of the courses and the HEIs. The performance evaluation of students in each course participating in ENADE is expressed through concepts, ordered on a scale with five levels^(^ 24 ^)^.

The results of the last assessment, carried out by the students show that 37.4% of the courses obtained grade 3, being also the predominant grade in the Southeast region (41.9%). When comparing the performance of public and private schools in the Southeast, it is observed that 34.37% of undergraduate public nursing courses obtained the ENADE 5 concept, while 44.36% of the private network courses achieved the ENADE 3 concept^(^ 24 ^)^.

In addition to the ENADE concept, another quality indicator of undergraduate courses evaluated by the Ministry of Education is the Preliminary Course Concept (*Conceito Preliminar de Curso*, CPC). This indicator is calculated based on the performance evaluation of students, on the value added by the training process and on inputs related to the supply conditions: teaching staff, infrastructure and didactic-pedagogical resources. The 2016 assessment data show that 50.5% of undergraduate nursing courses in Brazil obtained CPC 3^(^ 24 ^)^. Both grades obtained in national assessments coincide with the results of the ranking carried out in this research.

In Portugal, the quality assessment of undergra-duate is carried out by the Undergraduate Accreditation Education and Assessment Agency. All the training processes are subjected to *in loco* assessment cycles every six years, maintaining or not the authorization to operate according to the results obtained^(^ 25 ^)^.

This study brought adrift data that point to the non-compliance with the Brazilian educational legislation regarding the development of clinical education in the training courses for nurses. However, it should be noted that the universe of this research was limited to the state of São Paulo, Brazil, which, although it is the state with the largest number of undergraduate courses, may not portray nursing education in the country. It must be considered that the results were obtained through the report of the course coordinators, not being confirmed in other platforms, such as those available by the National Undergraduate Education Assessment System. Such findings reveal gaps in scientific knowledge on the subject, suggesting the possibility of further studies.

It is noteworthy that one of the main limitations of this study was the number of participants, since the population consisted of 168 nursing schools and 38 comprised the sample. Although there were several interventions (e-mail, telephone contact, presential meetings) in order to guarantee the greatest possible number of respondents, the delicacy of the research topic may have inhibited the achievement of a larger sample, especially among private schools, a 75% of the state’s Public Undergraduate courses participated in the survey. Therefore, such data do not allow information to be generalized, considering the continental characteristics of Brazil.

## Conclusion

The mean number of hours allocated to the ECS among the 38 nursing schools in São Paulo that comprised the study was 860.4 hours, preceded by 221.9 hours of simulated practices, and 508.1 hours of clinical practice in health services. The sum of hours for the ECS and the clinical practice corresponds to approximately 34% of the total hours of undergraduate courses. The scenarios in which the ECS has been carried out have proved to be diversified and serve the two main areas of health care in Brazil: primary and hospital care. The courses have used teaching methodologies in which the student is the center of the training processes and the assessment of learning.

Although the ECS is characterized by in-service teaching, in which the student participates in work processes guided by the figure of the nurse preceptor, and mediated by the didactic-pedagogical organization of the teacher, the student has been directly supervised by the teacher, with little participation by professionals that work in health establishments, internship fields, mischaracterizing the proposal. The mean of 3.1 points obtained identified in this study was similar to the results of national evaluations and show an average nursing education. The learning assessment processes proved to be important tools for obtaining teaching results.

The findings of this study point to the need to understand, in a qualitative way, *in loco*, how the nursing courses have effectively accomplished their curricular matrices, their course plans and complied with the educational legislation, because it is a profession that acts directly in the population’s health-disease processes and its results directly affect the Brazilian health system.
